# Three is a crowd

**DOI:** 10.1007/s12471-014-0586-0

**Published:** 2014-08-29

**Authors:** N. Lahrouchi, E. F. D. Wever, J. C. Balt

**Affiliations:** 1 Department of Experimental Cardiology, Academic Medical Center, University of Amsterdam, Meibergdreef 15, Room L2-109, 1105 AZ Amsterdam, the Netherlands; 2 Department of Cardiology, St Antonius Hospital, PO Box 2500, 3430 EM Nieuwegein, the Netherlands

## Answer to the rhythm puzzle

This patient has an arrhythmia with orthodromic circus movement tachycardia and dual AV-nodal pathways. In addition, the ECG shows a Brugada pattern.

The resting ECG (Fig. 1a) shows sinus rhythm and a type 2 Brugada pattern. The 12-lead ECG during arrhythmia (Fig. 1b) shows a narrow QRS rhythm, 88 bpm (cycle length: 680 ms) with a 1:1 AV ratio. The P waves are narrow and inverted in leads II, III, and aVF, and positive in aVR and V1 (superior P-wave axis), compatible with a septal origin. The RP interval is remarkably long (320 ms) and so is the PR interval (360 ms). These findings are consistent with orthodromic circus movement tachycardia (OCT) employing a slowly conducting retrograde accessory pathway (permanent junctional reciprocating tachycardia, PJRT) in combination with slow AV conduction over a putative slow pathway. The differential diagnosis includes atypical (slow–slow) atrioventricular nodal reentrant tachycardia (AVNRT) and atrial tachycardia with long AV-conduction (over a slow pathway).

**Fig. 2:**
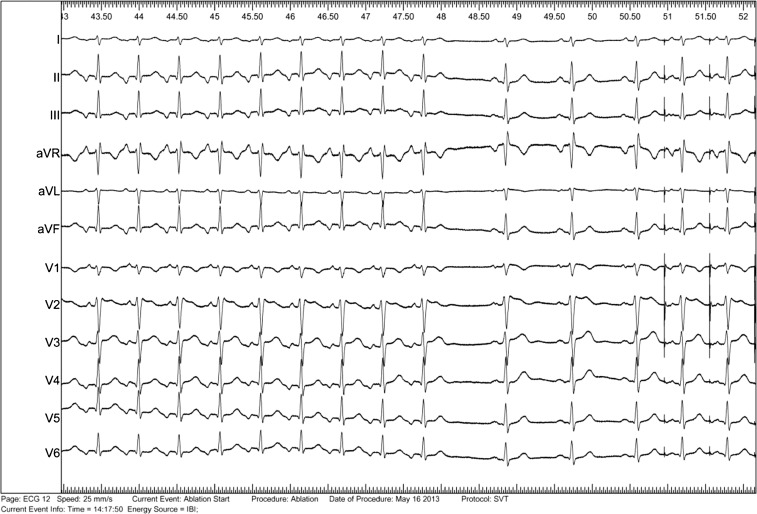
12-lead ECG demonstrating termination of the PJRT (with long RP but short PR signifying AV conduction over the fast pathway) with appearance of sinus rhythm after ablation of the accessory pathway. Thereafter, atrial pacing was started to assess atrioventricular conduction

An electrophysiological study was performed that confirmed the mechanism of the arrhythmia to be an OCT employing a slowly conducting retrograde accessory pathway. The existence of dual AV-nodal pathways was also confirmed. Depending on antegrade conduction over the slow or fast pathway, the cycle length of the arrhythmia was either 680 ms (88 bpm) or 510 ms (117 bpm). Radiofrequency ablation at the insertion of the accessory pathway in the proximal coronary sinus promptly terminated the tachycardia (Fig. 2).

**Fig. 3:**
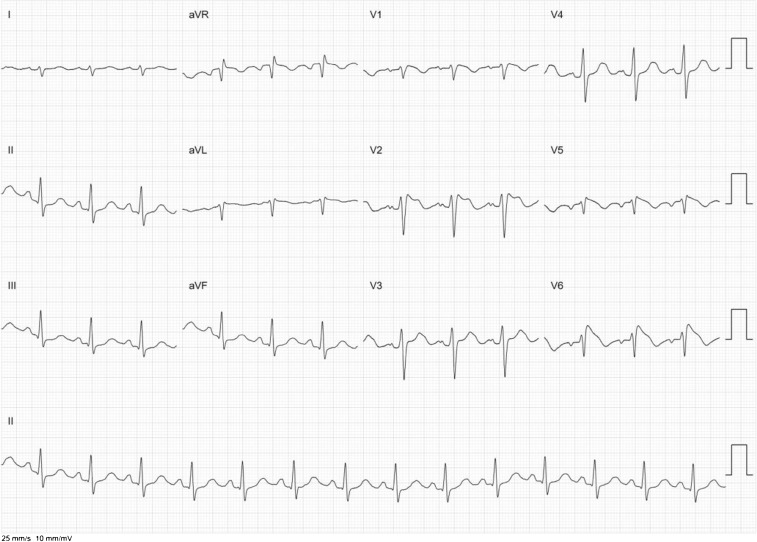
After infusion of 150 mg flecainide: 12-lead ECG with electrodes V5 en V6 positioned over the 3rd intercostal space, cranially from V1 and V2. Marked ST elevation is noted in V6 with a pattern that typically occurs in type I (coved-type) Brugada syndrome

Because of the type 2 Brugada ECG pattern, a provocative drug test with intravenous flecainide was performed which led to coved-type ST elevations in the precordial leads, i.e. a Brugada type I ECG (Fig. 3) ascertaining the diagnosis of Brugada syndrome [1].

PJRT is an uncommon (incessant) orthodromic tachycardia using a concealed, slowly conducting retrograde accessory pathway occurring predominantly in infants and children [2]. The surface 12-lead electrocardiogram of this narrow complex tachycardia is characterised by a long RP interval and inverted P waves in the inferior leads during tachycardia. The accessory pathway location is usually right posteroseptal with an atrial insertion nearby the ostium of the coronary sinus. This form of accessory-pathway-mediated tachycardia can lead to a tachycardia-induced cardiomyopathy and congestive heart failure if left untreated. Radiofrequency catheter ablation of the accessory pathway has become the treatment of first choice due to the unsatisfactory results of pharmacological therapy [3]. The patient was discharged in a good clinical condition and remains symptom free to date.
